# Scaling up Kangaroo Mother Care in Ethiopia and India: a multi-site implementation research study

**DOI:** 10.1136/bmjgh-2021-005905

**Published:** 2021-09-09

**Authors:** Prem K Mony, Henok Tadele, Abebe Gebremariam Gobezayehu, Grace J Chan, Aarti Kumar, Sarmila Mazumder, Selemawit Asfaw Beyene, Krishnamurthy Jayanna, Dejene Hailu Kassa, Hajira Amin Mohammed, Abiy Seifu Estifanos, Pankaj Kumar, Arun Singh Jadaun, Tedros Hailu Abay, Maryann Washington, Fitsum W/Gebriel, Lamesgin Alamineh, Addisalem Fikre, Alok Kumar, Sonia Trikha, Fisseha Ashebir Gebregizabher, Arin Kar, Selamawit Mengesha Bilal, Mulusew Lijalem Belew, Mesfin Kote Debere, Raghav Krishna, Suresh Kumar Dalpath, Samson Yohannes Amare, H L Mohan, Thomas Brune, Lynn M Sibley, Abraham Tariku, Arti Sahu, Tarun Kumar, Marta Yemane Hadush, Prabhu Deva Gowda, Khalid Aziz, Dereje Duguma, Pramod Kumar Singh, Gary L Darmstadt, Ramesh Agarwal, Dawit Seyoum Gebremariam, Jose Martines, Anayda Portela, Harsh Vardhan Jaiswal, Rajiv Bahl, Suman Rao PN, Birkneh Tilahun Tadesse, John N Cranmer, Damen Hailemariam, Vishwajeet Kumar, Nita Bhandari, Araya Abrha Medhanyie, KMC Scale-Up Study Group

**Affiliations:** 1 Division of Epidemiology & Population Health, St John's Medical College and Research Institute, Bangalore, India; 2 College of Health Sciences, Department of Paediatrics and Child Health, Addis Ababa University, Addis Ababa, Ethiopia; 3 Department of Pediatrics and Child Health, College of Medicine and Health Sciences, Hawassa University, Hawassa, Ethiopia; 4 Emory Ethiopia-Country Office, Addis Ababa, Ethiopia; 5 Boston Children’s Hospital, Boston, Massachusetts, USA; 6 Department of Pediatrics, Department of Epidemiology, Harvard Medical School, Boston, Massachusetts, USA; 7 Community Empowerment Lab, Lucknow, Uttar Pradesh, India; 8 Center for Health Research and Development, Society for Applied Studies, New Delhi, India; 9 School of Public Health, College of Health Sciences, Mekelle University, Mekelle, Ethiopia; 10 Karnataka Health Promotion Trust, Bangalore, India; 11 Ramaiah University of Applied Sciences, Bangalore, India; 12 College of Medicine and Health Sciences, School of Public Health, Hawassa University, Hawassa, Ethiopia; 13 School of Public Health, Addis Ababa University, Addis Ababa, Ethiopia; 14 National Health Mission, Government of Uttar Pradesh, Lucknow, Uttar Pradesh, India; 15 Department of Pediatrics and Child Health, School of Medicine, College of Health Sciences, Mekelle University, Mekelle, Ethiopia; 16 St John’s Research Institute, St John’s Medical College, Bangalore, India; 17 Emory Ethiopia-Amhara Regional Office, Bahir Dar, Ethiopia; 18 St. Paul’s Hospital Millennium Medical College, Addis Ababa, Ethiopia; 19 Governent of Uttar Pradesh, Lucknow, Uttar Pradesh, India; 20 State Health Systems Resource Center, Panchkula, Haryana, India; 21 Tigray Regional Health Bureau, Mekelle, Ethiopia; 22 Karnataka Health Promotion Trust, Rajajinagar, India; 23 School of Public Health, College of Medicine and Health Sciences, Hawassa University, Hawassa, Ethiopia; 24 Department of Software Engineering, School of Computing, College of Science and Technology, Mekelle University, Mekelle, Ethiopia; 25 Sachs’ Children and Youth Hospital, Stockholm, Sweden; 26 Emory University, Atlanta, Georgia, USA; 27 Maternal & Child Health Department, Federal Ministry of Health, Addis Ababa, Ethiopia; 28 Directorate of Health & Family Welfare Services, Government of Karnataka, Bangalore, India; 29 Department of Pediatrics, University of Alberta, Edmonton, Alberta, Canada; 30 Federal Ministry of Health, Addis Ababa, Ethiopia; 31 Department of Pediatrics, Stanford University School of Medicine, Stanford, California, USA; 32 Pediatrics, All India Institute of Medical Sciences, New Delhi, India; 33 Department of Maternal, Newborn, Child and Adolescent Health and Ageing, World Health Organization, Geneva, Switzerland; 34 Department of Neonatology, St John's Medical College Hospital, Bangalore, India; 35 Addis Ababa University, Addis Ababa, Ethiopia; 36 School of Public Health, Mekelle University College of Health Sciences, Mekelle, Ethiopia

**Keywords:** paediatrics, health systems, other study design

## Abstract

**Objectives:**

Kangaroo Mother Care (KMC), prolonged skin-to-skin care of the low birth weight baby with the mother plus exclusive breastfeeding reduces neonatal mortality. Global KMC coverage is low. This study was conducted to develop and evaluate context-adapted implementation models to achieve improved coverage.

**Design:**

This study used mixed-methods applying implementation science to develop an adaptable strategy to improve implementation. Formative research informed the initial model which was refined in three iterative cycles. The models included three components: (1) maximising access to KMC-implementing facilities, (2) ensuring KMC initiation and maintenance in facilities and (3) supporting continuation at home postdischarge.

**Participants:**

3804 infants of birth weight under 2000 g who survived the first 3 days, were available in the study area and whose mother resided in the study area.

**Main outcome measures:**

The primary outcomes were coverage of KMC during the 24 hours prior to discharge and at 7 days postdischarge.

**Results:**

Key barriers and solutions were identified for scaling up KMC. The resulting implementation model achieved high population-based coverage. KMC initiation reached 68%–86% of infants in Ethiopian sites and 87% in Indian sites. At discharge, KMC was provided to 68% of infants in Ethiopia and 55% in India. At 7 days postdischarge, KMC was provided to 53%–65% of infants in all sites, except Oromia (38%) and Karnataka (36%).

**Conclusions:**

This study shows how high coverage of KMC can be achieved using context-adapted models based on implementation science. They were supported by government leadership, health workers’ conviction that KMC is the standard of care, women’s and families’ acceptance of KMC, and changes in infrastructure, policy, skills and practice.

**Trial registration numbers:**

ISRCTN12286667; CTRI/2017/07/008988; NCT03098069; NCT03419416; NCT03506698.

**Key questions**

**What is already known?**

- Preterm births and low birth weight contribute to 80% of neonatal deaths.
- Kangaroo Mother Care (KMC) reduces mortality in stable babies <2000 g in hospital settings.
- WHO and national policies exist to support KMC, yet global coverage remains low.

**What are the new findings?**

- High population-based coverage of KMC can be achieved using a model derived through implementation research.
- The model includes strong government leadership, health workers’ conviction that KMC is the standard of care, women’s and families’ acceptance of KMC, and changes in infrastructure, policy and practice.
- Key to success are KMC units, ecosystems that keep mother and baby together, provide basic amenities and services for the mother–baby pair, effective counselling and technical support.

**Key questions**

**What do the new findings imply?**

- KMC implementation can be successfully scaled up across the hospital-to-home continuum of care.
- This study provides a KMC model that is adaptable for large scale implementation in different contexts.

## Introduction

Improving newborn survival is essential for achieving Sustainable Development Goal 3.2 (SDG-3.2).^1^ This would mean reducing the global neonatal mortality rate (NMR) from the 2017 rate of 18 deaths per 1000 live births in all countries to 12 deaths or fewer by 2030. More than 80% of neonatal deaths occur in low birth weight (LBW) infants.^2^ The United Nations Inter-Agency Group for Child Mortality Estimation indicated that, in 2018, the NMR in South Asia and in sub-Saharan Africa was 26 and 28 per 1000 live births, respectively; accounting for 79% of global newborn deaths. In addition, about three quarters of LBW births occur in these two regions.

While evidence is available on the effectiveness of interventions for newborn survival,^3^ similar evidence is often lacking on how to achieve high coverage of these interventions, and to maintain quality.^4^ Implementation research promises help in finding answers to why coverage and quality are low, what solutions may be effective, and how to apply them at scale in different contexts. Key interventions for preventing deaths among LBW newborns include Kangaroo Mother Care (KMC, defined as prolonged skin-to-skin care of the baby with the mother or other caregiver for as long as possible during day and night, and exclusive breastfeeding or breast milk feeding), antenatal corticosteroids for women with imminent preterm birth, and continuous positive airway pressure for preterm babies with respiratory distress.^5^ KMC has the potential to reduce mortality in LBW babies <2000 g by up to 40%.^6 7^ This can only happen if we achieve high-quality, universal coverage of KMC in the target population. Most countries have a policy of providing KMC to LBW babies, yet the estimated coverage of KMC globally is very low.^8 9^ Efforts are underway to identify effective strategies to increase KMC coverage.^10^

To this end implementation research was carried out in settings in Ethiopia and India, covering a combined population of 8 million people. The research objectives were to develop context-adapted implementation models and assess the achievement in coverage of KMC. We documented model development, implementation and programme performance. Settings had high numbers of LBW babies and NMR, and no alternative programmes for increasing KMC coverage.

## Methods

### Implementation science conceptual framework

We considered commonly used implementation science frameworks discussed in the systematic review by Moullin *et al*,^11^ including RE-AIM, PRISM and CFIR. While each of these frameworks had useful elements, no single framework was judged to be a good fit for our study. Instead, we used the Generic Implementation Framework proposed by Moullin *et al* 11 as a starting point to develop our methodology. This framework considers the non-linear and recursive nature of the implementation process as being foremost to implementation. At the centre of the framework is the innovation to be implemented, and surrounding the innovation are the contextual domains or levels of influence. Throughout the implementation process, there are factors, strategies and evaluations that will influence the course of implementation to be taken into account. We strongly believed that adapting implementation based on process learning and the concurrent evaluation of effective coverage was critical to achieving study objectives. We also included key concepts from the dynamic adaptation process proposed by Aarons *et al* 12 in our methodology.

Details of study methods have been published^13^ and are summarised below.

### Study design

This study used a mixed-methods design. We applied principles of implementation science to develop an adaptive strategy to help programme managers and health workers identify ways to improve implementation while maintaining fidelity to the evidence-based practice promoted. A partnership was established between state and district government health managers and a local research institution. They were challenged to develop an implementation strategy for reaching over 80% coverage of KMC from a baseline of virtually zero.

In the pre-implementation phase of formative research, we gathered data on components of the health system and the organisation of services, on care providers and the study population. Findings were used to inform the development of an initial implementation model, addressing providers’ skills, community acceptance, systems components and services. This was followed by implementation with iterative assessments of performance. Feedback was based on qualitative programme learning (managers, health providers and women/family responses) and quantitative data on KMC coverage. The main outcome was defined as ≥8 hours of skin-to-skin care over 24 hours, and exclusive breastfeeding or exclusive breast milk feeding. Feedback revealed needed refinements of the model, and rapid adaptation led to new cycles of implementation and review until the desired performance was reached.

Infants weighing <2000 g, born in geographically defined rural and semi-urban study areas (one district in each site in India, 3–5 *woredas* (districts) in each site in Ethiopia) were eligible for KMC. We promoted KMC initiation for eligible infants in health facilities after they were medically stable (defined as breathing and circulation not requiring continuous medical support and monitoring, and infant not subject to rapid and unexpected deterioration).^14^ This was complemented with accurate measurements of birth weight, referral of eligible infants born at home or in small facilities to KMC implementing facilities, and continuing KMC at home after discharge.

The final evaluation of KMC coverage was conducted at population level over a 9 to 12-month period. Given that current guidelines recommend that KMC only be initiated in hospital and that none of the hospitals in the study areas routinely implemented KMC at baseline, we judged that a baseline assessment of population-based coverage was not relevant for the evaluation. This information was confirmed by the health authorities in each site and supported by observations in the initial facility assessments. The establishment of a control group and consideration of secular trends were also deemed unnecessary given the near-zero baseline of KMC practice and that no alternative efforts to promote KMC in health facilities were under way in the study areas.

### Study sites

Improving newborn survival is a government priority in Ethiopia and India, and KMC is part of national guidelines. KMC coverage, however, is very low. In Ethiopia, 3–6 *woredas* in each of the four largest regions were selected by the federal government for implementation: Amhara, Oromia, Southern Nations, Nationalities and Peoples’ Region (SNNPR) and Tigray. In India, one district in three different states from different geographic regions with different levels of NMR were selected: Haryana (Sonipat district, NMR ~20/1000 live births), Karnataka (Koppal district, NMR ~25/1000 live births) and Uttar Pradesh (UP, Raebareli district, NMR ~30/1000 live births).^15^

KMC was introduced in Ethiopia in 1996 and became part of national guidelines in 2014. Constraints to expansion have been associated with lack of funding for training and supplies, lack of designated space for KMC in health facilities and staff assigned to support KMC.^16^

KMC was introduced in India in 1994 and became part of the national guidelines in 2014 as part of the Newborn Action Plan.^17^ Studies have indicated various challenges to implementation in India, including difficulties in the identification of LBW babies for KMC, limited health workers’ knowledge and skills to establish and support KMC, and lack of space and infrastructure in facilities for KMC provision. Studies indicate, nonetheless, positive responses from mothers to KMC and family support for providing KMC at home.^18–20^

### Study organisation and roles

The study was implemented in collaboration between the ministry of health and a research group at each of the sites. Each research group supported the ministry of health with three teams: (1) programme learning, (2) implementation support and (3) evaluation. The evaluation team, which measured the study outcomes, acted independently from the other teams. Panel S1 in online supplemental file 1 summarises the study organisation and the roles of the teams, including the ministry of health.

10.1136/bmjgh-2021-005905.supp1 Supplementary data

### Phases of study implementation

**Figure 1:**
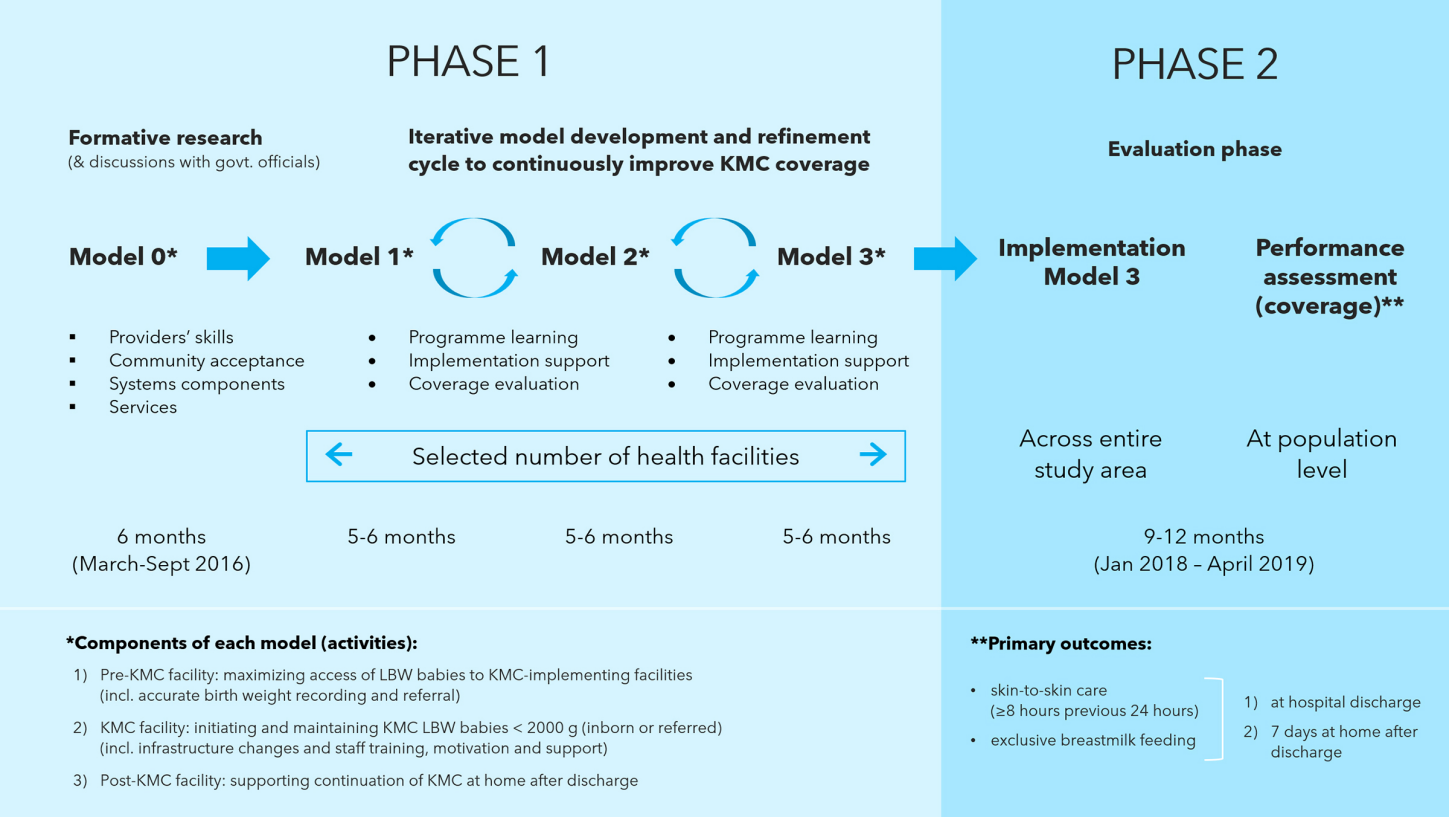
Overview of the study design and implementation. KMC, Kangaroo Mother Care; LBW, low birth weight.

Figure 1 provides an overview of the study design and implementation. In phase 1, we developed a KMC implementation model based on formative research and discussions at each site. Subsequently, there were cycles of implementation and refinement that led to models 1, 2 and 3 based on concurrent programme learning and coverage evaluation. Meetings of programme managers and researchers were conducted every 4–6 months to review implementation experience, information from programme learning and coverage data. Quantitative and qualitative information were triangulated to guide decisions. For example, information collected by the evaluation team and observations of programme staff on duration of skin-to-skin care were compared and correlated with the in-depth interviews with mothers conducted by the programme learning team. Using these inputs, refinements were proposed to the implementation model. During this process, in most sites, implementation was gradually expanded. Phase 2 consisted of implementing the final model 3 across the entire study area. Implementation continued to be conducted by the health system staff, with support from the research team. Performance was measured by the evaluation team from January 2018 to April 2019.

### Model components

Implementation models included three components: (1) *pre-KMC facility activities* aimed at maximising access of LBW babies to KMC-implementing facilities; these activities included accurate birth weight recording and referral of LBW infants born at home or in facilities that did not provide KMC. (2) *KMC-implementing facility activities* aimed at initiating and maintaining KMC for all LBW babies weighing <2000 g at birth who were born in or referred to the facility; these activities included changes in infrastructure and training, motivation and support of facility staff. (3) *Post KMC implementing facility activities* aimed to support the continuation of KMC at home after discharge. All research and implementation support materials developed can be found online (https://bit.ly/KMC-ScaleUp).

### Evaluation of final model performance

#### Participants

In the study area, all infants born in health facilities or the community within a 9 to 12-month evaluation period that ran, according to site, between January 2018 and April 2019, were screened by the evaluation team at regular visits to all facilities and community health workers. Infants with birth weight <2000 g whose mother was a resident of the study area, who had survived the first 3 days of age were eligible for inclusion in the evaluation.

### Outcomes

The primary outcomes were coverage of KMC (skin-to-skin care for ≥8 hours in the previous 24 hours and exclusive breast milk feeding) (1) at hospital discharge and (2) at 7 days after discharge in the home.

Additional outcomes included:

- Proportion of eligible infants who initiated KMC.
- Proportion of eligible infants receiving skin-to-skin care of any duration in 24 hours preceding discharge, at 7 days postdischarge and at 28 days of age.
- Duration of KMC, defined as the mean number of days of any skin-to-skin care during the neonatal period.
- Proportion of eligible infants exclusively breastfed (based on 24-hour recall) at discharge, 7 days postdischarge and 28 days of age.
- Neonatal deaths among babies with birth weight <2000 g whose mothers were residents of the study area.

The information on KMC duration and breastfeeding was based on mothers’ report.

The denominator for calculating KMC coverage was all eligible babies with birth weight <2000 g born in the study area to resident mothers; who did not die, did not leave the hospital against medical advice and were not referred to a facility outside the study area in the first 3 days and who were alive at the time of the outcome assessment.

### Sample size for final model performance assessment

We assumed that 3%–5% of newborns in Ethiopia and India would have a birth weight of <2000 g (based on facility record reviews) and that approximately 20% of them would not be assessed because of early death or loss to follow-up. We estimated we needed a minimum of 310 newborns per site to evaluate achievement of 80% coverage with site-specific absolute precision of at least ±5%.

### Ethical considerations

Individuals were not asked for consent to receive the intervention, as KMC was the government standard of care. Individual written informed consent was requested from mothers, caregivers, and health workers for the collection of study data. For those unable to read, the information was read by a team member in the presence of a witness who subsequently signed the consent form based on the individual’s decision.

Ethics approvals were received from the committees of the WHO and the participating research institutions. The results of the study have been disseminated and discussed with healthcare workers and managers in the different sites.

### Patient and public involvement

Through the iterative design process described earlier, mothers, carers, community members and health providers made important contributions to the study design and implementation.

### Role of the funding source

The funders of the study had no role in study design, data collection, data analysis, data interpretation or writing of the report.

## Results

Reporting has been guided by the Standards for Reporting Implementation Studies.^21^

### Study sites and population

Information on the study regions in Ethiopia and states in India is presented in panel S2 in online supplemental file 1. A substantial proportion (25%–54%) of mothers were illiterate. In the three states in India and in Tigray, most births occurred in public health facilities; in the three other regions in Ethiopia, home births were more frequent. Haryana (28%) and Karnataka (33%) in India had a substantial proportion of births in private facilities. NMR ranged from 34 to 47/1000 in the Ethiopia regions and from 18 to 45/1000 in the Indian states. Most mothers in the Indian states reported a postnatal check within 2 days of birth (59%–71%) but a substantially lower proportion did so in Ethiopia (9%–45%). The population across all study sites was about 8.7 million. The majority were rural, except for Tigray (44%). The proportion of LBW babies was higher in India (18%–26%) than in Ethiopia sites (12%–13%). The proportion of babies with birth weight <2000 g was about 3% in Ethiopia and 4%–6% in India. None of the facilities in the study sites were systematically implementing KMC at the time of study initiation and no other initiatives promoting KMC in health facilities were implemented in the study areas for the duration of the study.

### Implementation model development

#### Formative research findings

Prior to the study, mothers in health facilities were not encouraged to keep LBW babies in skin-to-skin care. Although breastfeeding was frequent, there was limited support and assistance to solve problems. Identification of LBW babies relied on spring scales in poor repair. In many peripheral facilities, LBW infants were identified based on the appearance of small size rather than measurement. Records showed heaping around 2000 g and 2500 g. Health staff, including nurses, were aware of KMC but considered it inferior to incubator care. KMC was recommended in national guidelines but not practiced in district and lower-level facilities. In crowded postnatal wards, lack of space and shortage of staff were additional obstacles to implementing KMC.

In general, mothers sought discharge as early as possible, often within 6–12 hours because of lack of privacy, poor hygiene, perceived disrespectful treatment, lack of food and poor night security. In India and Ethiopia, community workers (accredited social health activists (ASHAs) and health extension workers, respectively), although expected to make home visits to newborns after discharge, often failed to do so.

Community members usually had no experience with KMC. Some informants believed that it might not be feasible nor acceptable, given the extremes of heat, other demands on mothers’ time and that mothers may be weak and anaemic.

### Model development, implementation and optimisation

At each site, the initial model was prepared based on formative research and on discussions with government officials about policies, experiences and required resources. Aspects of the initial model were similar across sites: establishment of a designated space for KMC in facilities with a high number of births; staff trained and motivated to initiate and support KMC; accurate weighing of infants at birth in all health facilities and in the community to identify babies <2000 g; referral and transfer of babies to KMC-implementing facilities and support to continue KMC at home after discharge.

Models were implemented, assessed and refined during implementation (see panel S3 in online supplemental file 1 that presents the evolution from the initial to the final model in each site). Average KMC coverage at discharge was about 25% after the implementation of model 1, 40% after the implementation of model 2, and close to 55% when model 3 was in place. The main components of the final model common to all sites are presented in table 1 (and by health system building blocks in panel S4 in online supplemental file 1). Each site prepared implementation models adapted to its context. While the final model in table 1 shows the common components across sites, important differences between sites are summarised in table 2.

**Table 1:** Main components of the final model common to all sites

Pre-KMC-implementing facility	In KMC-implementing facility	Post KMC implementing facility
Birth weight for all babies born in non-KMC-implementing facilities accurately taken with digital scales and recorded by trained health workers (HWs), and birth weight of home births recorded by community health workers (CHWs). Referral of all <2000 g babies to a KMC-implementing facility assisted by HWs. HWs motivated, supported and monitored to perform above tasks. Community engaged to accept and support referral of newborns <2000 g for KMC.	Conducive environment for KMC established and maintained (facilities and staffing). Policies supportive of KMC established—mothers given rights and means to stay with babies (beds, food, bathing, toilet, etc). HWs motivated and supported to help mothers start and provide effective KMC. Counselling provided by HWs to sustain effective KMC while in the facility and after discharge. Birth weight of inborn babies accurately measured and recorded, and newborns <2000 g transferred to newborn intensive care unit or KMC ward. Performance of staff and facility conditions for KMC monitored and supported.	Links (eg, phone calls and referral slips) established between KMC facility and CHWs to inform about discharge of <2000 g babies. Home visits by CHWs held to support KMC at home after discharge from facility. Champions (such as experienced mothers) identified to promote and assist with KMC in the community. Community events held to talk about benefits of KMC—for example, health fairs, celebrations of 6-month/first birthday. Performance of CHWs in supporting KMC reviewed in regular supervision contacts.

KMC, Kangaroo Mother Care.

**Table 2:** Differences in content or implementation of the final model across different sites (vis a vis the ‘common’ model)

Site	Prefacility	Facility	Postfacility
Haryana	No major difference.	KMC implementation established at private facilities in addition to large public facilities. Dedicated family KMC area created outside the KMC ward. Additional KMC nurses deployed by the state government.	Families empowered to contact community health workers for home visits postdischarge.
Karnataka	No major difference.	KMC implementation established at private facilities in addition to large public facilities. On-site mentoring of staff by nurse mentors. Supportive visits by a team from a medical college. Skill-building via neonatal emergency drills/ perinatal audits. New mothers supported by experienced KMC mothers referred to as ‘AKKA’ chain (AKKA in local language refers to elder sister). KMC activities in postnatal wards.	Family level microplanning tool to help community health workers support KMC at homes and problem-solve. Animated videos in local television cable network/radio interviews/media.
Uttar Pradesh	Referrals to KMC-implementing facilities from private facilities promoted. Self-help groups encouraged referrals to KMC-implementing facilities.	Reclining chairs placed in special newborn care unit for intermittent KMC. Nurse coaches reviewed and improved nurse performance in KMC unit. Additional KMC nurses deployed by the state government. Data-driven monthly performance review by government. Interfacility social network of providers for sharing challenges, solutions and success stories. Planning for home transition with the mother/family at discharge (schedule for KMC at home, use of wrap/binder for ambulatory KMC, etc).	Helpline and counselling for KMC available 24×7. Baby-care teams including doctors and nurses made home visits for follow-up. Vouchers given to mothers for community health worker home visits.
Amhara	Champion mothers and their families (who benefited from KMC) facilitated early identification and referral of LBW babies at monthly meetings of pregnant women. Referral audit used to see the quality of referral services provided and clinical outcomes.	KMC cases from busy referral hospitals offloaded to primary hospitals. Peer education among KMC practicing mothers and families.	No major difference.
Oromia	Birth weight assessment and referral of <2000 g only in health facilities. Champion mothers to promote KMC in the community.	KMC promoted in labour and delivery wards and neonatal intensive care units, in addition to KMC units. Family integrated newborn care introduced in one of the sites. Expanded counselling and support team with staff and experienced mothers in addition to doctors and nurses.	No major difference.
SNNPR	Home birth identification and referral network strengthened.	Enhanced counselling support, audio-visual tools and mother support groups.	No major difference.
Tigray	Use of pregnancy cohort register for following pregnant mothers. Use of life event celebration in the community of KMC infants when they reach 6 months of age.	KMC provided in health centres, in addition to hospitals. Group counselling of mothers on KMC. KMC counselling using a checklist and supported by pictures and videos.	Use of two cards for postdischarge follow-up: one by health extension workers (HEW, community health worker) and the other by HEW supervisors.

KMC, Kangaroo Mother Care; LBW, low birth weight; SNNPR, Southern Nations, Nationalities and Peoples’ Region.

### Characteristics of the study population and performance of the final implementation model

The performance of the final model was assessed in a population of 3804 newborns. Data analysis was conducted using Stata V.16 (Stata Corp). All sites achieved or exceeded the estimated sample size (range 307–862), except SNNPR which enrolled 46% of the sample size in the period of evaluation. From 14.0% to 36.7% of mothers across sites had never been to school (table 3). The proportion of adolescent mothers was low. A higher proportion of eligible infants were reported as born at ≤8 months in Ethiopia (41.7%–75.2%) than in India (21.1%–45.9%). The proportion of very LBW (<1500 g) among eligible infants was higher in Ethiopia (20.5%–28.7%) than in India (12.1%–16.3%).

**Table 3:** Characteristics of infants and families included in the evaluation

	Amhara	Oromia	SNNPR	Tigray	Haryana	Karnataka	Uttar Pradesh
	N=603 (%)	N=307 (%)	N=143 (%)	N=424 (%)	N=762 (%)	N=703 (%)	N=862 (%)
Mother never been to school	221 (36.7)	60 (19.5)	20 (14.0)	62 (14.6)	108 (14.2)	131 (18.6)	191 (22.2)
Father never been to school	207 (34.3)	25 (8.1)	10 (7.0)	90 (21.2)	61 (8.0)	151 (21.5)	105 (12.2)
Adolescent mother <20 years of age	19 (3.2)	17 (5.5)	4 (2.8)	29 (6.8)	42 (5.5)	26 (3.7)	1 (0.1)
Reported gestation ≤8 months	270 (44.8)	231 (75.2)	76 (53.1)	177 (41.7)	318 (41.7)	148 (21.1)	396 (45.9)
Birth weight <1500 g	126 (20.9)	70 (22.8)	41 (28.7)	87 (20.5)	118 (15.5)	115 (16.3)	104 (12.1)
1500 – 1800 g	280 (46.4)	116 (37.8)	56 (39.2)	174 (41.0)	247 (32.4)	257 (36.6)	310 (36.0)
1800 – <2000 g	197 (32.7)	121 (39.4)	46 (32.2)	163 (38.4)	397 (52.1)	331 (47.1)	448 (52.0)

SNNPR, Southern Nations, Nationalities and Peoples’ Region.

KMC was initiated for 86.5%–87.4% of eligible infants in India and 67.7%–86.0% in Ethiopia (panel S5 in online supplemental file 1). In most sites KMC was initiated at a mean age of 4.4–7.1 days. Exceptions were UP, where KMC was initiated earlier (mean 1.2 days), and SNNPR, where it was initiated later (mean 9.7 days).

KMC with skin-to-skin care for ≥8 hours and exclusive breastfeeding in the 24-hour period before discharge (first primary outcome) was provided to 53.4%–82.3% of infants across sites. As shown in table 4, skin-to-skin care was provided during the 24 hours before discharge for 65.4%–89.2% of infants in the Ethiopia sites and 84.6% to 92.7% of infants in the India sites. Combined effective coverage of KMC was 68.1% in Ethiopia and 55.5% in India. Because many mothers in the Indian sites provided skin-to-skin care for fewer than 8 hours per day, coverage was lower. The mean number of hours of KMC in the 24-hour period before discharge ranged from 9.6 to 12.0 hours in India and from 11.6 to 14.9 hours in Ethiopia. Exclusive breastfeeding ranged between 63.8% and 88.5% across sites.

**Table 4:** KMC in the 24-hour period before discharge from facility

Site	Any skin-to-skin care, n (%)	Hours of skin-to- skin care per day (mean, SD)	>8 hours of skin-to-skin care, per day, n (%)	Exclusive breastfeeding, n (%)	KMC (>8 hours of skin-to-skin care and exclusive breastfeeding), n (%)
Amhara, n=602	394 (65.4)	14.9 (2.8)	391 (65.0)	384 (63.8)	380 (63.1)
Oromia, n=307	205 (66.8)	11.6 (4.0)	174 (56.7)	216 (70.4)	167 (54.4)
SNNPR, n=130	116 (89.2)	12.5 (4.0)	110 (84.6)	115 (88.5)	106 (81.5)
Tigray, n=384	329 (85.7)	13.4 (3.5)	323 (84.1)	333 (86.7)	316 (82.3)
Haryana, n=746	631 (84.6)	11.1 (4.7)	484 (64.9)	596 (79.9)	451 (60.5)
Karnataka, n=665	565 (85.0)	9.6 (4.4)	398 (59.8)	500 (75.0)	355 (53.4)
Uttar Pradesh, n=852	790 (92.7)	12.0 (7.9)	464 (54.5)	695 (81.6)	449 (52.7)

KMC, Kangaroo Mother Care; SNNPR, Southern Nations, Nationalities and Peoples’ Region.

At home, 7 days after discharge, skin-to-skin care continued for 60.2% of infants in Ethiopia and 78.3% of infants in India (table 5). Home-practiced KMC, with ≥8 hours of skin-to-skin care and exclusive breastfeeding in the previous 24 hours (second primary outcome) covered between 53.2% and 64.8% of infants across five sites, except Oromia (37.8%) and Karnataka (36.4%). Combined coverage of KMC at home with skin-to-skin care for ≥8 hours was slightly higher in India (55.2%) than in Ethiopia (52.2%). All sites had a mean duration of skin-to-skin care of more than 10 hours, except Karnataka (mean 8.0 hours). Exclusive breastfeeding ranged from 55.1% to 79.4% across sites.

**Table 5:** KMC at home 7 days postdischarge

Site	Any skin-to-skin care, n (%)	Hours of skin-to-skin care per day (mean, SD)	>8 hours of skin-to-skin care, per day, n (%)	Exclusive breastfeeding, n (%)	KMC (≥8 hours of skin-to-skin care and exclusive breastfeeding), n (%)
Amhara, n=594	332 (55.9)	11.5 (2.5)	323 (54.3)	327 (55.1)	316 (53.2)
Oromia, n=286	154 (53.8)	10.0 (3.8)	118 (41.3)	171 (60.0)	108 (37.8)
SNNPR, n=106	69 (65.1)	10.5 (5.0)	61 (57.5)	76 (71.7)	61 (57.5)
Tigray, n=362	256 (70.7)	12.0 (4.1)	227 (62.7)	255 (70.4)	218 (60.2)
Haryana, n=727	588 (80.9)	10.6 (4.2)	468 (64.4)	577 (79.4)	444 (61.1)
Karnataka, n=657	446 (67.9)	8.0 (3.1)	273 (41.6)	417 (63.5)	239 (36.4)
Uttar Pradesh, n=843	710 (84.2)	11.6 (4.3)	599 (71.1)	655 (77.7)	546 (64.8)

KMC, Kangaroo Mother Care; SNNPR, Southern Nations, Nationalities and Peoples’ Region.

At 28 days of age, the proportion of eligible infants receiving any skin-to-skin care ranged from 33.0% in Oromia to 79.2% in UP (see panel S5 in online supplemental file A). The mean duration of KMC ranged from 26 to 28 days in most sites. The proportion of infants exclusively breastfed at 28 days ranged from 53.0% in Amhara to 81.8% in Haryana. The coverage of exclusive breastfeeding across sites averaged 70.1%.

Of infants with birth weight <2000 g born to resident mothers, 16%–18% died within the neonatal period in all sites, except in UP (22.7%), Tigray (24.6%) and SNNPR (24.7%).

## Discussion

In seven sites with populations of 1–1.5 million, researchers and the government developed an implementation model for the local health system and achieved high-population coverage of KMC initiation. Overall, KMC was initiated for 82% of eligible infants, 60% received KMC for ≥8 hours on the day of discharge and 52% continued to receive KMC ≥8 hours per day at home 7 days postdischarge. The average duration of skin-to-skin care was 9.6–14.9 hours in the 24 hours prior to discharge.

The currently proposed coverage indicator for KMC in the Every Newborn Action Plan is ‘percentage of LBW newborns initiated on facility-based KMC’. We did not use this for KMC coverage because we believe that initiation is necessary but not sufficient for benefitting from KMC. Thus, we used coverage of ≥8 hours KMC at discharge from the facility, KMC is defined as continuous skin-to-skin care between the mother and the baby and exclusive breast milk feeding. However, intermittent KMC has shown similar benefits and the minimum hours of skin-to-skin care per day to achieve the benefits is not known. Previous studies indicated that skin-to-skin care for 7–8 hours or more per day is likely to be effective.[Bibr R15] 22 We therefore considered KMC as ≥8 hours of skin-to-skin care per day, in addition to exclusive breast milk feeding. It is noted that the average duration of skin-to-skin care in our study was similar to that achieved in a trial conducted by Mazumder *et al* in India, which reported a 30% reduction in neonatal mortality in the KMC group.^20^ [Bibr R16]

Some differences in performance across sites are noteworthy. Oromia, Karnataka and UP had the lowest proportion of newborns receiving KMC ≥8 hours per day at discharge. This is possibly due to the weaker health systems in these sites. In most sites, the proportion of newborns receiving KMC ≥8 hours per day was further reduced when assessed at home 7 days postdischarge, except for Haryana and UP. The strong support to sustaining KMC at home in these two sites through home visits by health workers and an active call centre (in UP) are likely to have played a significant role helping mothers and mobilising family support to provide more hours of skin-to-skin care after discharge.

The Generic Implementation Framework proposed by Moullin *et al* 11 related well to our study. The pre-implementation phase was important to prepare an initial implementation model. The strategies for overcoming barriers to implementation were informed by programme learning and concurrent evaluation in a recursive implementation process. Post-study implementation, the final model was scaled up to several non-study districts, particularly in India.

Several barriers related to each of the health systems have been identified in the literature associated with KMC implementation. They include lack of priority and leadership support within the health system, staff availability and training, inadequate resources and space allocation, as well as community acceptance.^8^ It is notable that acceptance by mothers and the community was not found to be an important barrier in our study. Other previously identified barriers were present in our study sites. The model described in this paper supported change by engaging health system managers and researchers to go beyond identifying problems but also work together in testing solutions within their specific setting. In this way, the process moved beyond the identification of barriers and facilitators to actively develop more appropriate scale-up models, transforming barriers into drivers of implementation and change.

Common elements for success were observed. The leadership and engagement of the government promoted supervision, health workers’ accountability and mobilisation of human and financial resources to cover large populations. This was facilitated by the alignment of project goals with the government priority of reducing neonatal mortality, country plans and, in the case of India, budget allocations for KMC. In Ethiopia and India, previous research on KMC^23–26^ may have enhanced the receptivity and support to KMC by the government partners. Supportive media highlighted the value of the work, sustaining motivation and rewarding decision-makers and health managers.

A key change was that the health system recognised the central role of the mother in newborn care, enabling mothers and babies to remain together. Facility management identified dedicated KMC space and provided for the mothers’ needs, including beds, chairs, food, amenities and hygiene. Success required convincing health workers about KMC benefits, strengthening their skills, and ensuring tools and resources for supporting KMC.

The institutionalisation of KMC was also reflected in the linkages developed between different units within the facility, different levels of facilities, and with the community for continuity of care. Systems for accountability and quality assurance were essential and proved the hardest to establish. The development of an information system for generating, analysing and using data for programme guidance was key to progress and will be essential for sustainability.

Engagement of mothers and families was fundamental. The novel organisation of services to allow the practice of KMC, with the mother staying in a room where other mothers practised KMC supported by health workers may have significantly facilitated adoption. Moreover, mothers’ experiences of providing critical elements of care such as body warmth and breast milk, and perceiving the baby’s response becoming warmer, active and growing appeared to support high adoption.

The final element for success was the empowering value of implementation research. Ambitious coverage targets were set, and implementation models were designed and adapted for use across large populations. Concurrent coverage assessment, programme learning and review of information by implementation teams helped increase motivation and action. The network of researchers and government jointly examining data and sharing experiences was synergistic in accelerating progress. The networking also helped to find solutions for important barriers, such as the absence of a KMC unit and need for advocacy for human and financial resources.

Several elements of this study make it highly relevant for global public health. It was implemented in two large countries in Africa and South Asia, the regions with most newborn deaths. We worked in seven sites, aiming to capture variability of contexts and challenges of implementing at scale, covering over 8 million people. Government leadership and engagement were assured from the beginning, with the request for proposals formulated and disseminated by the national ministries of health in collaboration with WHO. Site proposals were jointly developed by regional/state health managers and researchers from local institutions. The development and implementation of models was led by health managers relying on local research groups for initial implementation support, programme learning and evaluation. As the models should be feasible for dissemination to other settings, health managers were encouraged to think beyond the study sites. The success in this large-scale collaboration was encouraging. In the three states in India and in the Tigray region in Ethiopia, implementation of the KMC model extended beyond the study area even before the end of the project. In addition, investigators and the National Ministry of Health in Ethiopia obtained funding from the Global Financing Facility to expand KMC implementation using the study’s models.

Some potential limitations of this study merit consideration. First, we chose not to have a concomitant comparison arm because implementation of KMC in facilities was absent before the study and there was no competing intervention. In addition, the sample size reached in each of the sites would be sufficient to assess with high precision the coverage achieved with KMC. SNNPR was the only site not to achieve the planned sample size within the evaluation period because the majority of small babies admitted to KMC implementing facilities were from outside the study area.

Second, due to the study design, results cannot be attributed to individual components of the models. As shown in the presentation of the models, some approaches proved ineffective and were dropped; others were successful and preserved, sometimes with adaptations. A third possible limitation is that KMC coverage was based on mothers’ reporting. We acknowledge that both the Hawthorne effect and recall bias in reporting compliance are possible, particularly for reporting KMC 7 days postdischarge. Within proximity of implementation support and programme learning teams, social desirability bias cannot be excluded.

The study has important implications for newborn health programmes. We demonstrated with high plausibility that reaching high coverage with KMC implementation is possible, but we believe this can only be achieved with strong government leadership and commitment. We identified health system changes required for high KMC coverage including: (1) introducing a policy of zero separation between mother and baby, (2) maintaining infrastructure and practices allowing mothers a greater role in caring for their hospitalised children, including in neonatal care units and (3) providing integrated care that prioritises the needs of the mother and baby. However, system changes need tailoring to the implementation context. In some contexts, for example, where private providers are responsible for a substantial proportion of childbirth and newborn care, private sector engagement will be needed. Monitoring is critical to guide implementation and to maintaining stakeholders’ interest. Sustainability of KMC efforts is likely to be enhanced by integrating KMC-specific indicators in state and national health information systems.

We believe the adaptation and use at national scale of the models prepared in this study should contribute to reducing neonatal mortality and achieving the child mortality reduction target of SDG3. We also encourage future studies to apply a similar approach to implementation challenges for other difficult-to-scale public health interventions.

## Data Availability

Data are available upon reasonable request. Data are available upon reasonable request from the principal investigator of each site and/or the corresponding author.
