# Supplementary material for: Scaling up Kangaroo Mother Care in Ethiopia and India: a multi-site implementation research study
Source: BMJ Glob Health. 2021 Sep 9;6(9):e005905. doi: 10.1136/bmjgh-2021-005905 (PMC8438727; doi:10.1136/bmjgh-2021-005905)
Supplement: Supplementary data [file bmjgh-2021-005905supp001.pdf]

SUPPLEMENTARY MATERIAL A

Panel S1. STUDY ORGANISATION AND FUNCTIONS OF MINISTRY OF HEALTH AND RESEARCH GROUP

	**Ministry of Health**	**Research Group**		
Overall Role	Decision-making & implementation	Support for implementation and qualitative and quantitative data collection		
Staff	Programme managers and health facility staff	Programme learning team	Implementation support team	Evaluation team
Phase 1 activities	Deliver and monitor KMC intervention, take final decisions on the implementation model.	Conduct formative research to help develop the initial implementation model.<br><br>Collect and share qualitative data on why KMC was or was not being implemented. Contribute to discussions for refining the implementation models.	Support government for implementation of KMC. Contribute to discussions for refining the implementation models.	Collect quantitative data on coverage and quality to help assess performance of the implementation model in the catchment area. Contribute to discussions for refining the implementation models.
Phase 2 activities	Deliver and monitor the KMC intervention.	Monitor implementation process.	Support government for implementation of KMC.	Collect quantitative data on coverage and quality to help assess performance of the final implementation model in the entire study population.

Mony PK, *et al. BMJ Global Health* 2022; 6:e005905. doi: 10.1136/bmjgh-2021-005905

BMJ Publishing Group Limited (BMJ) disclaims all liability and responsibility arising from any reliance placed on this supplemental material which has been supplied by the author(s)

Panel S2. SUPPLEMENTARY INFORMATION ON REGIONS/STATES AND RESPECTIVE STUDY SITES'

	Ethiopia Regions				India States		
	**Amhara**	**Oromia**	**SNNPR**	**Tigray**	**Haryana**	**Karnataka**	UP
**Illiterate mothers**	54% *	51% *	43.9% *	43% *	24.6% +	28.3% +	39.0% +
**No ANC visit**	32.4% *	48.6% *	30.4% *	9.6% *	17.1% +	11.0% +	23.7% +
**Home births**	71.4% *	80.5% *	72.5% *	41.0% *	19.6% +	6.0% +	32.2% +
**Private facility birth**	0.5% *	0.8% *	0.3% *	0.3% *	28.4% +	32.8% +	23.3% +
**Postnatal check of mother first 2 days**	18.4% *	9.0% *	16.9% *	45.4% *	70.8% +	65.8% +	58.8% +
**NMR**	47/1000 *	37/1000 *	35/1000 *	34/1000 *	22.1/1000 +	18.5/1000 +	45.1/1000 +
**IMR**	67/1000 *	60/1000 *	65/1000 *	43/1000 *	32.8/1000 +	26.9/1000 +	63.5/1000 +
	**Study Woredas**	**Study Woredas**	**Study Woredas**	**Study Woredas**	**Sonipat District**	**Koppal District**	**Raebareli District**
**Size (sq km)**	5,052	4,236	1,866	4,233	2,260	7,000	2,003
**Population (n) of study area**	1·5 million	1·1 million	1·1 million	0·6 million	1·5 million	1·5 million	1·4 million
**Study area rural**	70%	52%	67%	44%	70%	82%	100%
**KMC-implementing facilities**	2 teaching & 3 district hospitals	1 teaching, 2 general, 2 primary & 2 private hospitals	1 teaching, 2 general and 1 district hospitals	1 teaching, 5 general & 1 primary hospitals; 21 health centres	1 teaching, 1 district, 4 subdistrict & 4 private hospitals	1 district, 3 subdistrict, 4 private hospitals	1 district hospital, 9 subdistrict hospitals
**Expected live births/yr**	52,000	39,000	30,000	19,000	25,000	27,000	30,000
**Expected LBW <2500 g babies/yr**	6,000	5,100	4,000	2,400	4,500	7,000	7,200
**Expected <2000 g babies/yr**	1,500	1,200	900	600	1,000	1,500	1,500

Mony PK, *et al. BMJ Global Health* 2022; 6:e005905. doi: 10.1136/bmjgh-2021-005905

*Ethiopian Demographic and Health Survey 2016 (https://dhsprogram.com/pubs/pdf/FR328/FR328.pdf)

+ India National Family Health Survey (NFHS-4) 2015-2016 (https://dhsprogram.com/pubs/pdf/FR339/FR339.pdf)

Mony PK, *et al. BMJ Global Health* 2022; 6:e005905. doi: 10.1136/bmjgh-2021-005905

Panel S3. – ADAPTATION OF THE MODEL – WHAT WAS DROPPED, ADDED OR MODIFIED BETWEEN INITIAL AND FINAL MODELS

Site	Pre-facility	Facility	Post-facility
**Haryana**	***Dropped***<br>-Nothing<br><br>***Added***<br>-Digital scales (and training to use them) at all facilities.<br>-District KMC network created with private non KMC-implementing facilities<br><br>***Modified***<br>-KMC incorporated into routine behaviour change communication activities.<br>-Pregnancy surveillance strategy to include migrant population.<br>-Weight criteria for referral for KMC modified from <1800g to <2000g	***Dropped***<br>-Nothing<br><br>***Added***<br>-Services in newly established KMC wards improved, e.g. provision of cooked food, additional discharge criteria, daily counselling, discharge counselling);<br>-Dedicated KMC nurses recruited,<br>-Family KMC area created to increase family participation<br>-KMC model adapted for private hospitals;<br>-KMC indicators incorporated into HMIS as reportable items;<br><br>***Modified***<br>-KMC sessions started in SNCU.<br>-KMC initiated in postnatal wards.<br>-KMC integrated with essential newborn care.	***Dropped***<br>-Home contact by SNCU staff nurses and CHW (ASHA) coordinators<br><br>***Added***<br>-Supervisory committee responsible for field supervision<br><br>***Modified***<br>-Nothing
**Karnataka**	***Dropped***<br>-Village level health, sanitation and nutrition committees' engagement discontinued due to inadequate response<br>-Pregnancy surveillance dropped (resource intensive)<br><br>***Added***<br>-Supportive supervision by project staff along with ASHA facilitators<br><br>***Modified***<br>-Nothing	***Dropped***<br>-Handover of newborn from facility to CHW at discharge as this was not feasible to operationalize<br>-Phone call from facility to CHW at discharge<br><br>***Added***<br>-Continuing medical and nursing education and training for counsellors followed by onsite support visits by a team of experienced clinicians and nurses<br>- Neonatal emergency drills/ perinatal audits<br>-Stepping up KMC activities in postnatal wards<br><br>***Modified***<br>-Frequency of specialist mentoring reduced	***Dropped***<br>-PHC level review meetings<br><br>***Added***<br>-Link card issued by the KMC facility to the CHW through the family at discharge<br>-Supportive supervision to CHWs<br>-CHW champions<br><br>***Modified***<br>-CHW home visits on alternative days after discharge instead of daily

Mony PK, *et al. BMJ Global Health* 2022; 6:e005905. doi: 10.1136/bmjgh-2021-005905

Site	Pre-facility	Facility	Post-facility
**Uttar Pradesh**	***Dropped***<br>-Nothing<br><br>***Added***<br>-Performance recognition for CHWs<br><br>***Modified***<br>-Nothing	***Dropped***<br>-Nothing<br><br>***Added***<br>-Labour room provides SSC at birth<br>-KMC during referral transport & coordination between facilities to ensure preparedness<br>-Scientific communication materials and orientation for doctors<br>-Online videos and contextualised job aid and posters as counselling support tools<br>-Nurse coaches<br>-Data driven monthly review by Government<br>-Social network of providers sharing challenges, solutions and success stories among facilities<br>-HMIS for facility level KMC reporting<br>-Planning for home transition with mother/family at discharge.<br><br>***Modified***<br>-Nothing	***Dropped***<br>-Nothing<br><br>***Added***<br>-Baby care teams to make home visits<br>-Call centre 24X7 with counselling nudge for KMC<br><br>***Modified***<br>-Nothing
**Amhara**	***Dropped***<br>-Integrate key KMC messages into the family health guide (not feasible)<br><br>***Added***<br>-Biannual review meeting with key Health Centre staff<br>-Use HEW birth notification register to capture home births<br>-Use <2000gm baby tracking register at Health Centres to document referral for KMC<br>-KMC counselling during ANC<br>-Conduct referral audit<br>-Establish phone call contact Health Centre-Hospital for referral tracking system<br><br>***Modified***<br>-Nothing	***Dropped***<br>-Facilitate internal supportive supervision by NICU/KMC team at least on monthly basis (not feasible)<br><br>***Added***<br>-Continued training for newly assigned nurses of NICU/KMC and delivery units<br>-Participatory supportive supervision to strengthen linkage between labour and delivery ward, NICU and KMC unit<br>-NICU and KMC unit clinical audits<br>-Offload cases from referral hospital to primary hospital<br>-Make the setting of KMC units similar to home environment (coffee ceremony)<br>-Use of a KMC wrap in hospital and encouraged mothers to prepare their own at home<br><br>***Modified***<br>-Nothing	***Dropped***<br>-Nothing<br><br>***Added***<br>-Counsel families to assist the mother during KMC practice at home<br>-Link discharged babies from KMC with HEW through use of notification cards<br>-Provide referral feedback from hospital to health centres<br><br>***Modified***<br>-Nothing

Mony PK, *et al. BMJ Global Health* 2022; 6:e005905. doi: 10.1136/bmjgh-2021-005905

Site	Pre-facility	Facility	Post-facility
**Oromia**	***Dropped***<br>-CHW identification and referral of <2000g babies<br><br>***Added***<br>-Nothing<br><br>***Modified***<br>-Nothing	***Dropped***<br>-Implementation of KMC at Health Centres<br>-Scorecard to evaluate care of <2000g babies<br>-KMC review in hospital administrative meetings<br><br>***Added***<br>-Promotion of hand-hygiene of mothers and HWs in NICU and KMC Unit<br>-Newborn care focal person/Champion established<br><br>***Modified***<br>-KMC promoted in labour and delivery wards and NICU in addition to KMC ward<br>-Support staff and senior mothers included in supporting counselling, in addition to the nurses	***Dropped***<br>-Nothing<br><br>***Added***<br>-KMC Champion mothers to support home KMC<br><br>***Modified***<br>-Nothing
**SNNPR**	***Dropped***<br>-Use of Health Development Army<br>-Incentives for Health Extension Workers<br><br>***Added***<br>-Use of HMIS reporting format for KMC activities<br><br>***Modified***<br>-Nothing	***Dropped***<br>-Nothing<br><br>***Added***<br>-Implement intermittent KMC at NICU<br>-Newborn care Quality Improvement team established in the Hospital<br>-Family training using dolls for KMC position and danger signs<br>-Postnatal card for the mother at discharge<br><br>***Modified***<br>-Improve communication between labour and delivery ward, NICU and KMC unit<br>-Improve counselling using aids and ensuring privacy	***Dropped***<br>-Nothing<br><br>***Added***<br>-Nothing<br><br>***Modified***<br>-Hospital staff telephoned mothers after discharge to encourage KMC at home

**Site**	**Pre-facility**	**Facility**	**Post-facility**
**Tigray**	***Dropped***<br>-Pregnancy and birth tracing by *Woreda* level staff (not feasible)<br>-HEW weighing home delivered babies (not feasible)<br><br>***Added***<br>-Awareness creation using life event celebrations<br>-Information on KMC during ANC<br><br>***Modified***<br>-Strengthen pregnancy & birth tracing by HEW using pregnancy cohort form<br>-Engaging HEW to refer home delivered babies for weighing and assessment	***Dropped***<br>-Nothing<br><br>***Added***<br>-KMC group education<br><br>***Modified***<br>-Nothing	***Dropped***<br>-Nothing<br><br>***Added***<br>-Catchment based supportive supervision<br>-Use local radio station and TV channel to promote KMC<br><br>***Modified***<br>-Nothing

Mony PK, *et al. BMJ Global Health* 2022; 6:e005905. doi: 10.1136/bmjgh-2021-005905

Panel S4. KMC FINAL IMPLEMENTATION MODEL AND HEALTH SYSTEM BUILDING BLOCKS

**Policy, governance and leadership**	KMC implementation already policy of the ministries of health in Ethiopia and India and present in national strategies and newborn management guidelines. In India, policy was clarified to include hospital care of newborns <2000 g. State/Province and leadership engaged from the start in review of KMC coverage and improving performance.
**Service delivery**	KMC services were initiated with the study. Initially limited to the study facilities, it expanded to other public facilities in and outside of the study districts and to private facilities as the study progressed in India. Community structures such as village health and sanitation committees (at village level) and client welfare committees (facility level) were sensitized and oriented toward KMC/neonatal health.
**Health system financing**	In Ethiopia, there were no specifically allocated resources for KMC activities within health facilities. During the study, it was negotiated with the facilities to designate physical space and furniture. Facility leadership budgeted for KMC care in next years' budget. In India, funds for facilities and additional human resources were obtained provisionally and then included in new Annual Planning process (Project Implementation Plans – PIP) - at district and state levels. Also, in India, incentives were provided by the government to CHW for home visits for <2000gm babies and refer small newborn infants for screening.
**Health work force**	At study initiation, facilities did not have assigned personnel for KMC activities and CHWs lacked knowledge and skill to support on KMC. In both countries training was provided at each level of the health system. At study facilities, a focal KMC point person from within the existing HR structure was identified to support linkages between the health facility and community.
**Health information system**	KMC-specific indicators incorporated into data systems. Registries and clinical logs developed to track small babies in health facilities and district and used in reporting to district health authorities.
**Health infrastructure and supplies**	Space to house mothers together with the newborns was unavailable or inadequate. Facility management and district authorities helped identify or supported building space for KMC units and contributed resources to furnish and equip them, including chairs, ensuring availability of bathrooms and showers, TV screens, as well as production of communication tools and job-aids.

Mony PK, *et al. BMJ Global Health* 2022; 6:e005905. doi: 10.1136/bmjgh-2021-005905

Panel S5. INITIATION OF KMC AND KMC AT HOME AT 28 DAYS OF LIFE

INITIATION OF KMC

Site	KMC initiated n (%)
**Amhara, n=603**	408 (67.7%)
**Oromia, n=307**	245 (80.1%)
**SNNPR, n=143**	123 (86.0%)
**Tigray, n=424**	354 (83.5%)
**Haryana, n=762**	662 (86.9%)
**Karnataka, n=705**	616 (87.4%)
**UP, n=862**	746 (86.5%)

KMC AT HOME AT 28 DAYS OF LIFE

Site	Any skin-to-skin care, n (%)	Exclusive breastfeeding, n (%)
**Amhara, n=541**	345 (63.8%)	287 (53.0%)
**Oromia, n=282**	93 (33.0%)	214 (75.9%)
**SNNPR, n=128**	80 (62.5%)	88 (68.8%)
**Tigray, n=379**	236 (62.3%)	251 (66.2%)
**Haryana, n=727**	477 (65.6%)	595 (81.8%)
**Karnataka, n=644**	366 (56.7%)	382 (59.1%)
**UP, n=797**	631 (79.2%)	636 (79.8%)

Mony PK, *et al. BMJ Global Health* 2022; 6:e005905. doi: 10.1136/bmjgh-2021-005905

BMJ Publishing Group Limited (BMJ) disclaims all liability and responsibility arising from any reliance placed on this supplemental material which has been supplied by the author(s)

Mony PK, *et al. BMJ Global Health* 2022; 6:e005905. doi: 10.1136/bmjgh-2021-005905
